# Guidelines to manage women with infertility: an e-Delphi study

**DOI:** 10.4314/ahs.v24i3.31

**Published:** 2024-09

**Authors:** Armah Deborah, Annatjie van der Wath, Mariatha Yazbek, Florence Naab

**Affiliations:** 1 Department of Maternal and Child Health, School of Nursing, College of Health Sciences, University of Ghana, Ghana; 2 Department of Nursing Science, University of Pretoria, Pretoria, South Africa

**Keywords:** Holistic approach, e-Delphi technique, psychosocial interventions, infertility

## Abstract

**Introduction:**

The psychosocial health problems associated with infertility is compounded in African women when prevailing cultural expectations emphasize the value of parenthood. Despite the social stigma these women endure, they are still mostly managed from a biomedical approach, especially in Ghana, the context of the study.

**Objective:**

This study explored the views of a panel of experts to reach consensus on holistic guidelines to manage women with infertility in Ghana.

**Methods:**

An e-Delphi technique was employed to retrieve information using a panel of 20 experts. Data collection was conducted in two rounds, and each participating expert was emailed the preliminary guidelines to rate based on certain criteria. Data analysis was done in accordance with the expert participants' rating of and comments on the guidelines.

**Results:**

The final guidelines for holistic healthcare to manage women with infertility include the following interventions: Holistic healthcare assessment, psychological interventions, health education, spiritual support, relevant support from significant others; all within a therapeutic relationship.

**Conclusion:**

When healthcare providers use the final set of guidelines to manage women diagnosed with infertility, these women will receive holistic healthcare to attain optimal health and improved chances of conceiving.

## Introduction

Despite great success in improving maternal and child health in the past few decades, many people in low-and-middle-income countries do not have equal access to fertility care that is rarely seen as a priority in national universal health coverage benefit packages.^1^ In countries where resources are scarce, women have the least access to fertility care, while they are at the same time exposed to social rejection and emotional heartbreak.^2^,^3^

One in every seven couples in the western world, and one in every four in developing countries are estimated to be affected by infertility. Male infertility is solely responsible for 20–30% of infertility cases, and contributes to 50% of cases overall.^4^ While the estimated proportion of infertility causes are equally shared between men and women^5^, the psychological burden of infertility is mostly affecting women.^6^

Infertility is often accompanied by other health and mental health problems. Research indicated an increase in rates of depression, anxiety and psychosocial distress in women with infertility.^7^ Infertility is experienced as a crisis with biopsychosocial effects that influence all aspects of life.^8^,^9^ The importance attached to parenthood in Africa significantly contributes to the overall burden of infertility.^10^ Although the challenges faced by African women with infertility may differ from county to country, the mistreatment of these women cuts across most of the African society. The consequences of infertility include stigmatization, divorce, polygamy, extramarital relationships, disinheritance, physical and psychological abuse; compounded by limited access to information and fertility services.^12^ A study done in sub-Sharan Africa indicated a disproportionate social impact of infertility on women where a high value is placed on children and women with infertility are stigmatized.^13^

The social consequences of the stigma African women with infertility endure, include marital strains, potentially leading to separation or divorce, and exacerbation of psychological distress such as anxiety and low self-esteem. Recommendations to relief the psychosocial stress of infertility include advocacy, community mobilisation, health education, provision of holistic person-centered care^14^ and psychological interventions especially for African women who suffer social stigmatization.^15^

In spite of all the negative influences that infertility has on the wellbeing of women with infertility in Ghana, an Africa country known to be a strong pro-natal society^16^, women with infertility are mostly managed biomedically, making their management incomplete.^17^

The authors of this article conducted a study to develop guidelines for holistic healthcare for women with infertility in Ghana. In the first phase, the healthcare needs of women with infertility were explored through focus groups, secondly, the opinions of healthcare providers regarding healthcare interventions for women with infertility were obtained through a nominal group technique. The aim of the last phase, on which this article is based, was to the refine the guidelines to address the healthcare needs of women diagnosed with infertility in a holistic manner.

## Methods

### Study design

A consensus method was used to refine the guidelines to manage women with infertility in a holistic way. A Delphi technique is a method for obtaining judgements and views from an expert panel about a topic of interest with the agenda of seeking consensus on the issue. The e-Delphi technique is an online method that uses a multistage self-completed questionnaire with individual feedback to determine consensus from a larger group of experts.^18^ A set of draft guidelines developed from empirical data obtained from women with infertility and healthcare providers, was e-mailed to experts with a questionnaire to refine the guidelines.

### Study population and sampling

Forty-five expert participants who met the inclusion criteria were invited via e-mail to participate in the refinement of the guidelines. Inclusion criteria included: Both international and local experts from government and nongovernmental organizations in the area of infertility, policy and guideline development. Experts included; academic researchers, gynecologists, midwives, and nurse practitioners.

The researcher used purposive sampling to select the experts based on their research expertise and the specified inclusion criteria. Twenty expert participants responded to the email invitation. Between 10 and 18 experts are recommended for an e-Delphi panel^19^, therefore the researcher deemed 20 participants a sufficient sample

### Data collection procedures

A cover letter and informed consent form were sent to participants together with the draft guidelines and an instrument to evaluate the guidelines. The criteria used in the instrument to rate the guidelines were adapted from the guiding attributes for guideline development, reporting and evaluation as set out in the AGREE II document.^20^ The criteria included, scope of the guidelines, purpose of the guidelines, stakeholders' involvement, reliability, validity, clarity and applicability. Participants indicated the degree to which they agree or disagree that the guidelines met the criteria, using a 4-point Likert scale: Strongly disagree = 1, disagree = 2, agree = 3, strongly agree = 4. Participants were asked to note comments and recommendations at the end of the questionnaire. Data was obtained from the panel of experts in two rounds. The researchers facilitated the process of data collection in each round with a summary of the panel's views circulatng between rounds to achieve consensus.^18^

In Round 1 participants were given an overview of the draft guidelines based on a biopsychosocial spiritual model and the empirical findings. The participants rated the guidelines in accordance with the criteria as set out above and recorded their comments to refine the guidelines. Responses from the participants were screened, analyzed and collated. The consensus rate for each guideline was calculated as presented in Table 2.

In Round 2 the adjusted guidelines were re-sent to the participants after the necessary modifications were made for their final comments, if they deemed it necessary. This round gave the participants the opportunity to observe how their opinions differed from others, and an opportunity to generate additional insight and comments.

### Data analysis

Data analysis was done in accordance with the expert participants' rating of the guidelines. The number of responses were recorded according to the levels of agreement using the 4-point Likert scale. A quality score was calculated for each of the seven criteria according to AGREE II.^20^ Scores were calculated by summing up all the scores of the expert participant items and by scaling the total as a percentage of the maximum possible score. See Table 2. The participants' comments were read and categorized according to three themes.

### Rigour

To ensure trustworthiness, participants provided their opinions anonymously and independently without being influenced by anyone. Data was triangulated by using participants from different disciplines and contexts. Authenticity was obtained by using direct quotations from participants in the results. The guidelines were rated for reliability and validity by the expert panel.

### Ethical considerations

The study received ethical approval from the Ethics committee of the Faculty of Health Sciences, University of Pretoria. (Ethics Reference No.: 579/2018). Participation was voluntary and participants signed informed consent forms that they returned with the completed questionnaires. Participants were assigned numbers to ensure confidentiality. All responses were kept anonymous.

## Results

Table 1 outlines the demographic profile of the participants. The participants represented experts in infertility care and research from different countries.

**Table 1:** Descriptive information of the e-Delphi expert panel

No.	Professional qualification	Occupation	Country	Experience,
**1.**	PhD Nursing; Masters (Advanced Psychiatric Nursing); Bachelor of Nursing; Diploma in Nursing, Psychiatry, Community and Midwifery	Lecturer	South Africa	Head of Department: Midwifery
**2.**	Masters in Nursing; Bachelor of Nursing; Diploma in Nursing; Postgraduate Diploma in Education	Lecturer	Nigeria	Clinical practice: Ante-natal clinics, delivery suite, obstetrics, gynecology and family planning units
**3.**	Masters in Nursing; Bachelor of Nursing; Diploma in Nursing; Postgraduate Diploma in Education	Lecturer	Ghana	Academic researcher
**4.**	PhD candidate; Masters (Advanced Midwifery & Neonatology); Bachelor of Nursing & Midwifery; Postgraduate Diploma in Education	Lecturer	South Africa	Clinical midwife: High risk obstetric unit; lecturer undergraduate, post graduate and post basic midwifery
**5.**	Masters in Nursing; Bachelor of Nursing; Diploma in Nursing	Nurse/Lecturer	Ghana	Nurse; academic researcher
**6.**	Trainee in Obstetrics & Gynecology and Oncology	Obstetrician & Gynecologist	Indonesia	Obstetrician and Gynecologist
**7.**	Masters in Midwifery; Bachelor of Nursing; Diploma in Midwifery	Midwife	Ghana	Midwife
**8.**	Bachelor of Nursing; Diploma in Midwifery	Midwife	Ghana	Midwife
**9.**	PhD Nursing; Masters in Nursing; Diploma in Nursing and Midwifery; Public Health Nurse	Lecturer	Ghana	Clinical practice: Maternity and gynecology; academic researcher in female infertility
**10.**	Masters in Nursing; Bachelor of Nursing; Certificate in Nursing & Midwifery	Nurse/Midwife	Ghana	Nurse/Midwife
**11.**	Trainee in Obstetrics and Gynecology	Obstetrician & Gynecologist	Indonesia	Obstetrician and Gynecologist
**12.**	Trainee in Obstetrics and Gynecology	Gynecologist	Indonesia	Gynecologist
**13.**	MBChB; BSc	Medical Officer/Gynecologist	Ghana	Clinician department: Obstetrics and gynecology
**14.**	PhD Candidate; Masters in Nursing; Bachelor of Nursing; Diploma in Nursing	Lecturer	Ghana	Academic researcher in infertility
**15.**	PhD Psychology; MPhil Psychology; BSc Psychology	Lecturer	Ghana	Academic researcher
**16.**	Trainee in Obstetrics & Gynecology	Gynecologist	Indonesia	Gynecologist
**17.**	Masters in Nursing; Bachelor of Nursing; Diploma in Education; Certificate in Midwifery & Nursing	Lecturer	Ghana	Academic researcher in infertility; clinical practice in nursing and midwifery; infertility counseling
**18.**	Masters in Nursing	Midwife/Lecturer	Ghana	Academic researcher
**19.**	Masters in Nursing	Nursing/Midwife	Ghana	Midwife; researcher in maternal and child health
**20.**	PhD candidate; Masters in Nursing; Bachelor of Nursing; Diploma in Nursing	Lecturer	Ghana	Academic researcher

An overall consensus rate of 85% was obtained in round 1. According to AGREE II, high quality guidelines are those with consensus scores >70%.20 Table 2 indicates the consensus rates for the different criteria.

**Table 2:** Consensus rates of Delphi round 1 (n=20)

Guideline number	Guideline Scope of the guidelines				Purpose of the guidelines				Stakeholders' involvement				Reliability			
	**1**	**2**	**3**	**4**	**1**	**2**	**3**	**4**	**1**	**2**	**3**	**4**	**1**	**2**	**3**	**4**
**1**			21	52			27	44			27	44			18	56
**2**			21	52			33	36			24	48			24	42
**3**			18	56			30	40			24	48			21	52
**4**			30	40			24	48			27	44	1		24	44
**5**		2	15	56		2	33	36			27	44		1	15	56
**6**			21	52			30	40			24	48		4	24	40
**7**		2	18	52		2	27	44			33	36		1	27	40
**Total**		**4**	**144**	**360**		**4**	**204**	**288**			**186**	**312**	**1**	**6**	**153**	**330**
**Total**	**508**				**496**				**498**				**490**			
**Consensus rate**	**88%**				**85%**				**85%**				**83%**			

Guideline number	Validity				Clarity				Applicability			
	**1**	**2**	**3**	**4**	**1**	**2**	**3**	**4**	**1**	**2**	**3**	**4**
**1**			30	40			27	44			21	52
**2**			18	56			21	52		2	9	64
**3**		2	18	52			18	56		2	21	48
**4**		2	33	32	1		27	40	1	2	24	40
**5**		2	18	52		2	15	56		2	15	56
**6**		4	18	48		2	21	48		2	15	56
**7**		1	21	48		2	12	60		2	21	48
**Total**		**11**	**156**	**328**	**1**	**6**	**141**	**356**	**1**	**12**	**126**	**364**
**Total**	**495**				**504**				**503**			
**Consensus rate**	**85%**				**87%**				**86%**			

Three themes emerged from the comments of the expert panel, namely, psychoeducational aspects of the guidelines, spiritual and social support recommended in the guidelines. The panel members' comments are indicated in italics.

### Theme 1: Psychoeducational aspects of the guidelines

The experts indicated that …psychological interventions are fundamental as far as issues of infertility is concerned. The psychological interventions provided could help reduce the psychological burden of infertility since the mindset of the woman is key to positive outcomes in management…that will increase their chances of conceiving. The participants advised that psychological interventions should be provided within a therapeutic relationship where healthcare providers …recognise their cultural beliefs or practices and treat each woman as a unique individual. They suggested for healthcare providers: Avail yourself to the women by ‘being there’ and making time to listen to the women's concerns…respect the women's choice of support. The experts also suggested a relationship based on honesty and openness where healthcare providers need to … acknowledge that women diagnosed with infertility may sometimes require assistance with particular support or service that the healthcare provider is unable to deliver. Firstly, assess the availability and accessibility of that support or services. Healthcare providers need to indicate to the woman with infertility the boundaries of the relationship and clarify treatment expectations.

The experts indicated the empowering value of health education, when it will be implemented by women diagnosed with infertility, it will allow them take charge of whatever they are going through, whereas they will become responsible and independent. They recommend the development of …reading material (for example, pamphlets) for those who can read so that they can learn about various treatment options.

### Theme 2: Spiritual support recommended in the guidelines

Since the initial guidelines focused more on provision of spiritual interventions from a Christian background, the experts indicated that spiritual interventions should be formulated in such a way that it …include other religious backgrounds as well. Women may have diverse religious affiliations; hence this must be putting into consideration anytime there is the need to render such intervention. They also recommended healthcare providers to involve families when giving spiritual support and guidance since infertility is a major family concern.

### Theme 3: Social support recommended in the guidelines

The guidelines included social interventions and the experts agreed that …getting financial support will really relieve women diagnosed with infertility from lots of financial burdens since the cost involved in the management of infertility is overwhelming. They recommended to …include nongovernmental organizations, specifically those in charge of women's health, in financial assistance.

Since the research context was women with infertility, the initial guidelines did not include the partners, but the experts felt …that the partners of the women with infertility should be included in psychoeducational interventions, health education sessions and the therapeutic relationship.

## General comments from the panel of experts

The panel rated the guidelines as being aligned with the stakeholders included in the first phases of the study. The women with infertility indicated their healthcare needs as follows: A need for medical health assessment, psychological care, provision of health education regarding infertility, spiritual support, social support and financial support. They further indicated that they expect from healthcare providers to meet the mentioned needs, ensure privacy and confidentiality, and continuity of care. Women diagnosed with infertility wished to have a healing and supportive relationships with their healthcare providers. The healthcare providers recommended medical, psychological, spiritual and social interventions and the establishment of therapeutic relationships with women with infertility.

The panel also indicated that concepts must be used consistently in the guidelines, for example, either use problems or needs, preferably needs, and women or couple seeking care for fertility.

As an acceptable consensus rate was obtained in round 1, the researchers adapted the guidelines as recommended by the panel of experts. The seven adapted guidelines were distributed again to the experts so that they could see the changes made and add final comments if necessary. No additional comments were received in round 2.

## Discussion

The final guidelines with recommended actions are presented in Table 3. A holistic approach to fertility assessment includes a complete medical, reproductive, and family history and also, a thorough assessment of the woman's physical, psychological, cultural, social and spiritual needs.^21^,^22^ Holistic healthcare assessment reveals the actual cause of the problem and narrows the focus of diagnostic evaluation.^23^ To assess the nursing needs of women with infertility, a nursing needs assessment scale can be used.^24^ The scale measures 18 items distributed among four factors: physical and psychological nursing needs, needs for information on treatment, needs for infertility-related understanding and concern, and supportive needs.^24^

**Table 3:** Guidelines for holistic management of women with infertility

Guidelines	Actions
Conduct a holistic healthcare assessment to identify women's health support needs.	Ask critical questions to ensure all information is obtained and recorded Conduct a comprehensive physical examination to detect physical health needs Request appropriate investigations and obtain results before commencement of treatment.
Incorporate psychological interventions in the management protocols.	Provide, or refer as indicated: Psychological counselling, peer mentoring, cognitive behavioural therapy, acceptance commitment therapy, body-mind intervention and emotionally focused interventions. Involve partners and families in psychological care.
Provide health education about causes, prevention, various treatment options and side effects.	Assess prior knowledge to determine educational needs. Use language that is understood, refrain from medical terms or jargon. Provide information while assessing readiness to learn. Advise on lifestyle changes related to infertility. Use verbal, written, or visual aids and models that can be easily understood Evaluate uptake of information and additional needs.
Acknowledge spiritual needs and ensure the provision of spiritual support.	Respect spirituality as unique to women's belief systems Maintain a non-judgmental attitude Assess spiritual needs and refer as needed Encourage spiritual empowerment. Refer for spiritual support. Facilitate hope, meaning and purpose in life Address issues related to suffering and grief
Ensure relevant support from significant others.	Encourage the involvement of partners throughout the treatment Refer for couple and family support and therapy. Encourage mobilization of support from trusted family members. Respect women's choice of support.
Encourage the women to have their health insured and lobby for financial support from government and nongovernmental organizations.	Encourage women to enroll in health insurance schemes Ensure reproductive healthcare is accessible and affordable, based on the needs of women. Collaborate with private agencies and philanthropists to mobilize resources
Create a therapeutic relationship with the women and their partners in a therapeutic environment in order to meet healthcare needs.	Establish rapport, show respect and empathy Ensure privacy and confidentiality Provide adequate time for consultations. Avoid judgmental statements. Use effective verbal and non-verbal communication skills. Listen attentively and maintain eye contact during consultations. Acknowledge individuality. Offer additional support, for example, home visits.

Incorporation of psychological interventions in the management protocols was agreed upon by the panel of experts as the second guideline. The Fertility Life Counselling Aid is a step by step intervention designed for clinical staff to support patients with the psychological trauma associated with infertility in resource poor settings. This intervention is based upon cognitive behavioral therapy (CBT) strategies, and fosters holistic infertility care.^25^ Women with infertility preferred behavioral interventions and were willing to practice coping skills and mindfulness to help manage the uncertainty and stress of infertility.^26^ Mindfulness meditation improves emotional well-being and outcomes in infertility.^27^ Group psychological interventions, (based on CBT and mindfulness) improved the mental health, fertility stress and pregnancy rates of women undergoing fertility treatment.^28^

The third guideline is based on the need for health education in the management protocol of women with infertility. Equal access to quality health education and lifelong learning is fundamental to health promotion.^29^ Health education should be based on long-term, well-resourced and planned programmes, opposed to short-term, reactive and opportunistic interventions.^30^

Education to promote a healthy lifestyle, was effective in reducing the lifestyle risk factors for infertility and increasing the success rates of fertility treatment.^31^ Health education helps to decrease negative attitudes towards infertility and ensure culturally competent care.^32^ Information about the relationship between stress and infertility facilitated understanding of how stress and infertility interrelates, and encouraged the use of stress reduction techniques.^33^

The provision of spiritual support is described in the fourth guideline. Participants agreed that incorporating spirituality in healthcare may facilitate mental and physical wellbeing. Spirituality can influence the way people understand health, their resilience, health outcomes and sense of support.^34^ Spiritual care may contribute to healing and help patients and their family members to explore meaning in real-life situations.^35^ While religious beliefs and rituals can serve as coping mechanisms, for example, finding comfort in viewing infertility as part of a divine plan, healthcare providers should be aware that couples may perceive infertility as punishment from a higher power for sins and indiscretions.^36^

Ensuring relevant support from significant others was the fifth guideline refined by the panel. Support from significant others may help to alleviate the stress of infertility through encouragement, advice and reassurance to allay concerns and fears.^37^ Women supported by their families and especially their partners, are less susceptible to mental health problems such as depression.^38^ Women with high levels of perceived social support tend to use active-confrontational and meaning-based coping strategies, aimed at finding a solution. Advocacy is needed to ensure better support for women with infertility and their partners.^39^

Guideline six included lobbying for financial support from government and nongovernmental organizations. The financial burden of infertility plays a role in patients discontinuing their treatment regimen.^40^ Financial support for infertility treatment is better in high and middle-income countries than in low and middle-income countries, where preventive and supportive care services are neglected. To achieve equity in infertility care services, achieving reproductive goals should be seen as a universal human right.^41^

The final guideline recommends that healthcare providers should create a therapeutic relationship with women and their partners in order to meet their healthcare needs. One of the ways to create a therapeutic healthcare environment is to follow a holistic and patient-centered approach when providing care for infertility to enhance quality of life and wellbeing.^42^ Providers should be mindful of their behavior and attitudes to align with what patients value about the relationship.^43^ A therapeutic relationship requires a caring and authentic presence, instills hope; and is characterized by compassion, respect and trust.^44^

## Study limitations

The research relied on the responses of the invited e-Delphi experts representing only a few countries and practice environments. Wider representation could have created more input and perhaps more recommendations for the guidelines. The guidelines are however based on the data obtained from women with infertility and practice nurses in the research context.

## Conclusion

The holistic management of women diagnosed with infertility contributes to the success of treatment and overall well-being. The guidelines can be adapted for different healthcare contexts and management protocols to include holistic healthcare assessment, psychological interventions, health education, acknowledgement of spiritual needs, involvement of significant others and promotion of financial support. All these interventions should take place within the context of a therapeutic relationship.
